# Supplementary figures and images for: Global circRNA expression changes predate clinical and histological improvements of psoriasis patients upon secukinumab treatment
Source: PLoS One. 2022 Sep 29;17(9):e0275219. doi: 10.1371/journal.pone.0275219 (PMC9522259; doi:10.1371/journal.pone.0275219)

A

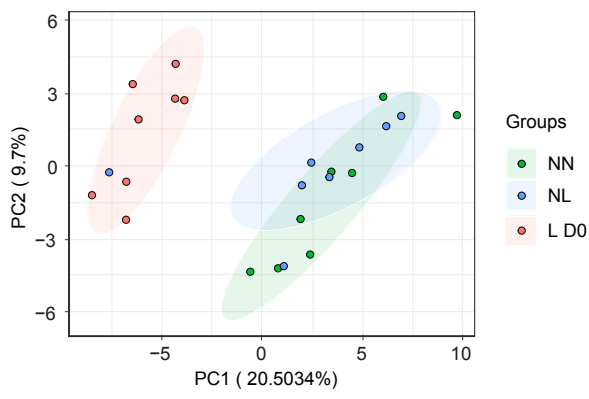

B

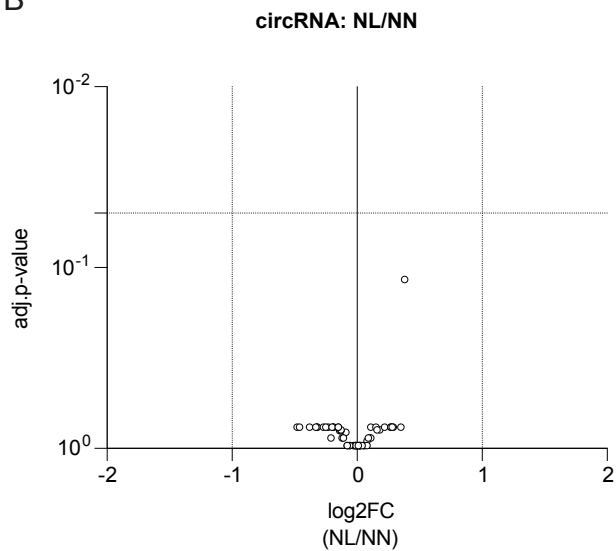

C

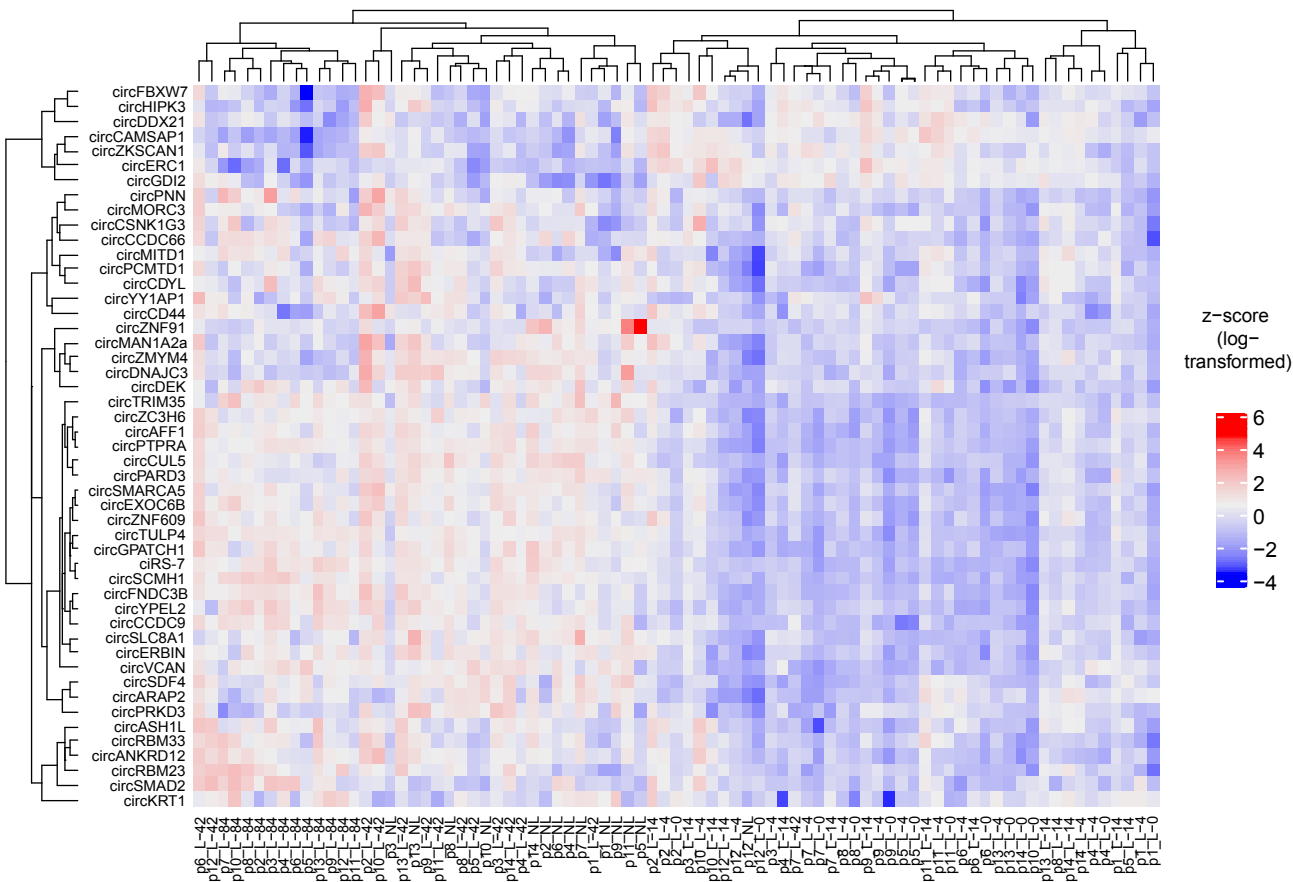

D

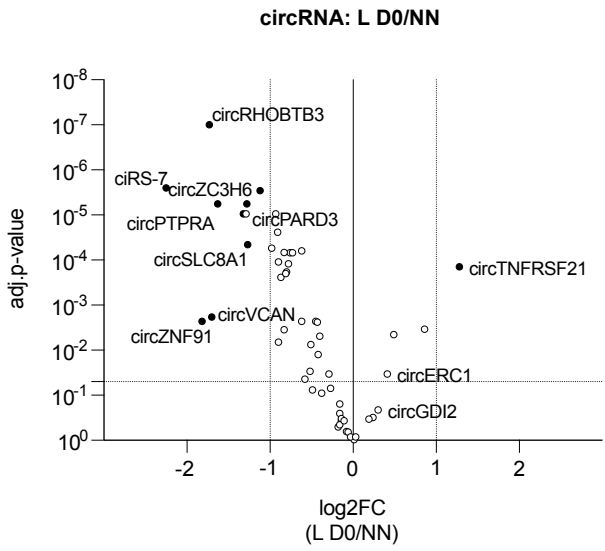

E

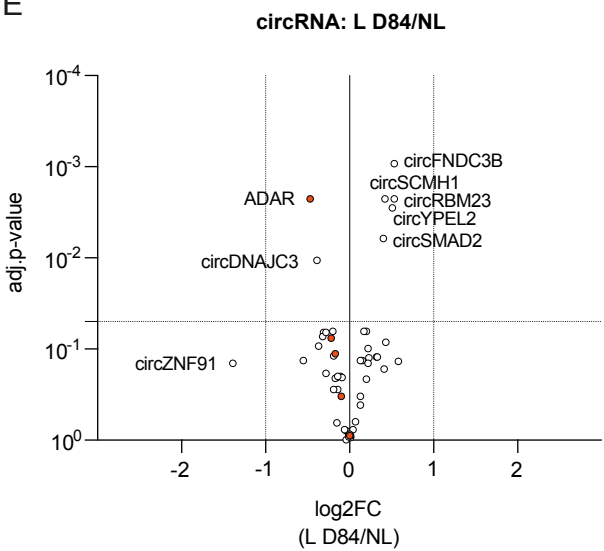

Figure S1

Supplement: S1 Fig — (A) Principal component analysis based on circRNA expression levels in paired lesional and non-lesional patients before treatment and healthy control skin (NN). (B) Volcano plot showing changes circRNA expression in non-lesional psoriasis skin before treatment relative to healthy control skin (NN). Plots depict adjusted (adj.) p-values relative to log2FC. Multiple unpaired t-test with correction for multiple comparisons (FDR-Benjamini-Hochberg). (C) Heatmap with unsupervised hierarchical clustering of circRNA expression (as z-score of log-transformed values) for individual patients from non-lesional and paired lesional psoriasis skin dependent on the day of treatment. (D) Volcano plot showing changes circRNA expression in lesional psoriasis skin before treatment relative to healthy control skin (NN). Plots depict adjusted (adj.) p-values relative to log2FC. Multiple unpaired t-test with correction for multiple comparisons (FDR-Benjamini-Hochberg). (E) Volcano plot showing changes in specific circRNA and mRNA expression in lesional psoriasis skin at day 84 of treatment (L D84) compared to non-lesional skin. Multiple paired t-test with correction for multiple comparisons (FDR-Benjamini-Hochberg). For panels C and E: n(NL, L D4, L D14, and L D43) = 14, n(L D0) = 13, and n(L D84) = 12; for panels A, B, and D: n = 8. (PDF) [file pone.0275219.s001.pdf]

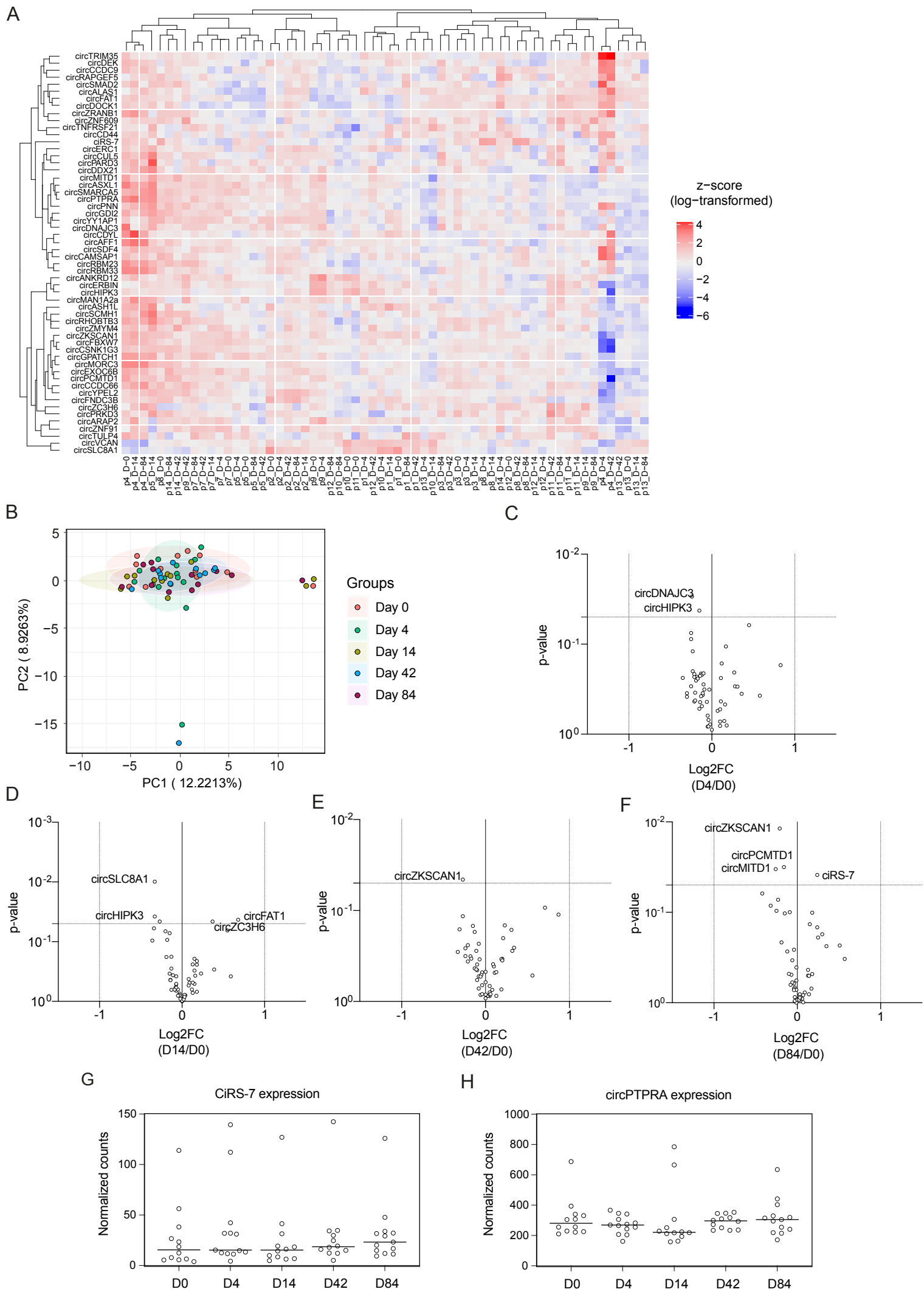

**Figure S2**

Supplement: S2 Fig — (A) Heatmap using unsupervised hierarchical clustering of circRNA expression levels (as z-score of log-transformed values) in peripheral blood mononuclear cells (PBMCs) from patients before (D0; n = 12) and after 4 (D4, n = 13), 14 (D14; n = 12), 42 (D42; n = 12), and 84 (D84; n = 13) days of treatment. (B) Principal component analysis based on circRNA expression levels in peripheral blood mononuclear cells (PBMCs) from patients before (D0) and after 4 (D4), 14 (D14), 42 (D42), and 84 (D84) days of treatment. (C-F) Volcano plot showing changes in circRNA expression in PBMCs from patients at day four (D4; C), day 14 (D14; D), (D42; E), and day 84 (D84; F) in contrast to day 0 (D0) of secukinumab treatment. Depicted are p-values relative to the log2FC. Multiple paired t-test without correction for multiple comparisons. (G-H) CiRS-7 (G) and circPTPRA (H) expression in PBMCs during 84 days of secukinumab treatment. Depicted are normalized counts and median expression. One-way ANOVA with correction for multiple comparisons (FDR-Benjamini-Hochberg); n(D0, D14, D42) = 12 and n(D4, D84) = 13. (PDF) [file pone.0275219.s002.pdf]

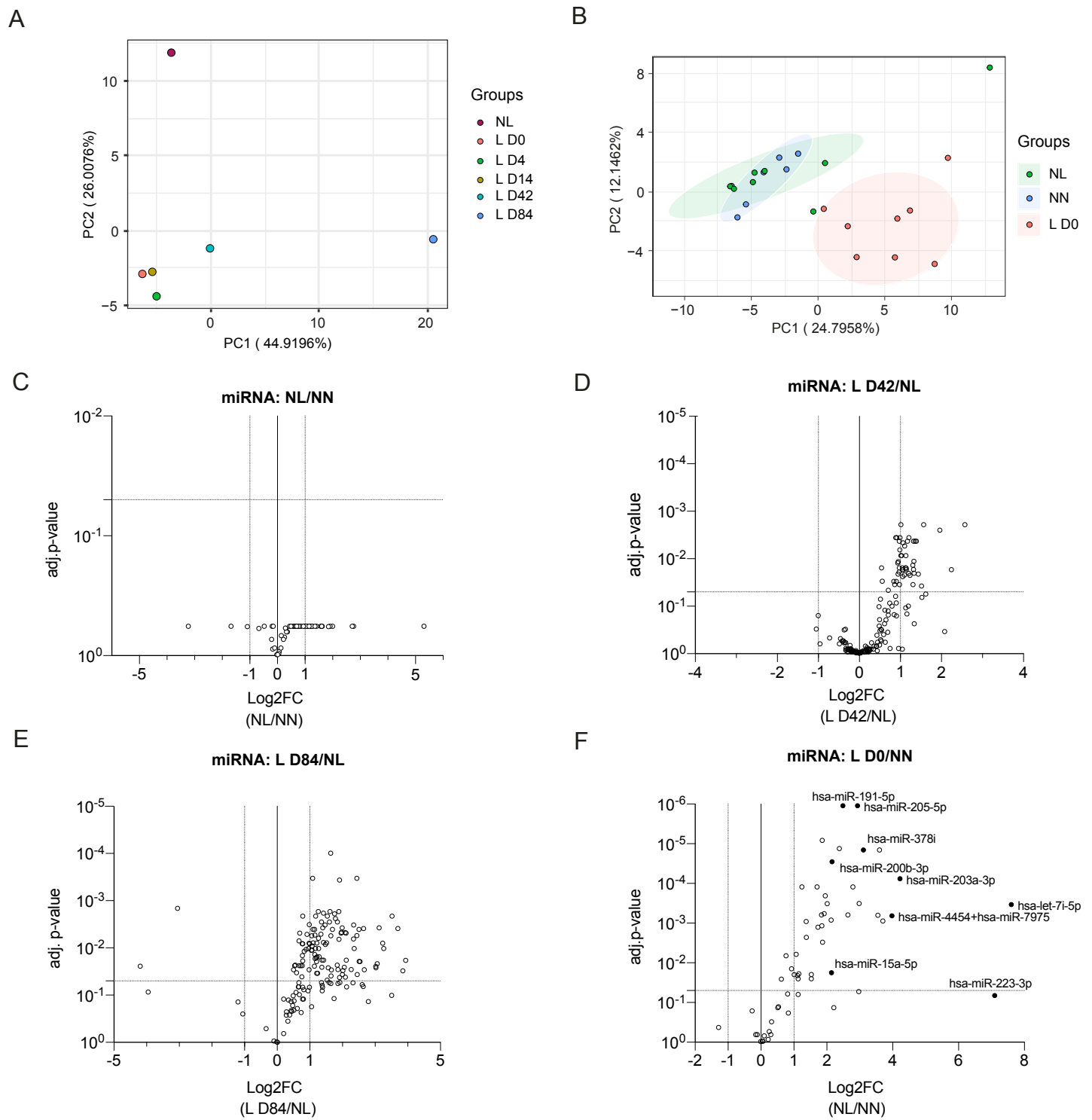

Figure S3

Supplement: S3 Fig — (A) Principal component analysis based on mean miRNA expression levels between patients from non-lesional and paired lesional psoriasis skin before (L D0) and after 4 (L D4), 14 (L D14), 42 (L D42), and 84 (L D84) days of treatment. (B) Principal component analysis based on miRNA expression levels in paired lesional and non-lesional patients before treatment and healthy control skin (NN). (C) Volcano plot showing changes miRNA expression in non-lesional psoriasis skin before treatment relative to healthy control skin (NN). Plots depict adjusted (adj.) p-values relative to log2FC. Multiple unpaired t-test with correction for multiple comparisons (FDR-Benjamini-Hochberg). (D-E) Volcano plot showing changes in miRNA expression in day 42 (D) and 84 samples (E) in contrast to non-lesional skin. Multiple paired t-test with correction for multiple comparisons (FDR-Benjamini-Hochberg). (F) Volcano plot showing changes miRNA expression in lesional psoriasis skin before treatment relative to healthy control skin (NN). Plots depict adjusted (adj.) p-values relative to log2FC. Multiple unpaired t-test with correction for multiple comparisons (FDR-Benjamini-Hochberg). For panels A, D, and E: n(NL, L D4, L D14, and L D43) = 14, n(L D0 and L D84) = 13; for panels B, C, and F: n(NL, L) = 8 and n(NN) = 7. Depicted are miRNAs with an average expression above 20 counts (n = 58). (PDF) [file pone.0275219.s003.pdf]

A

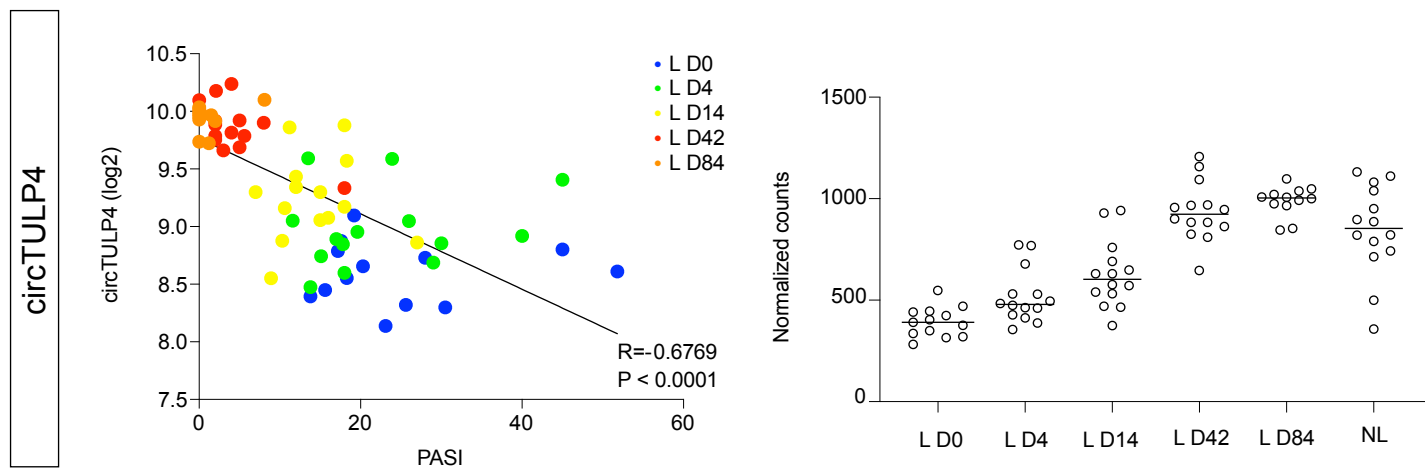

B

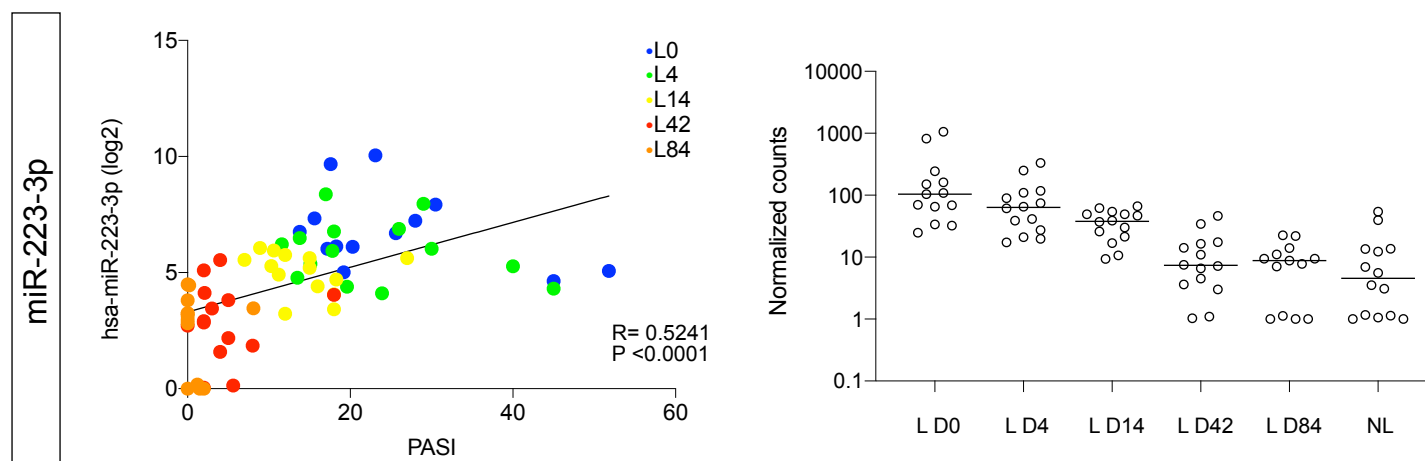

C

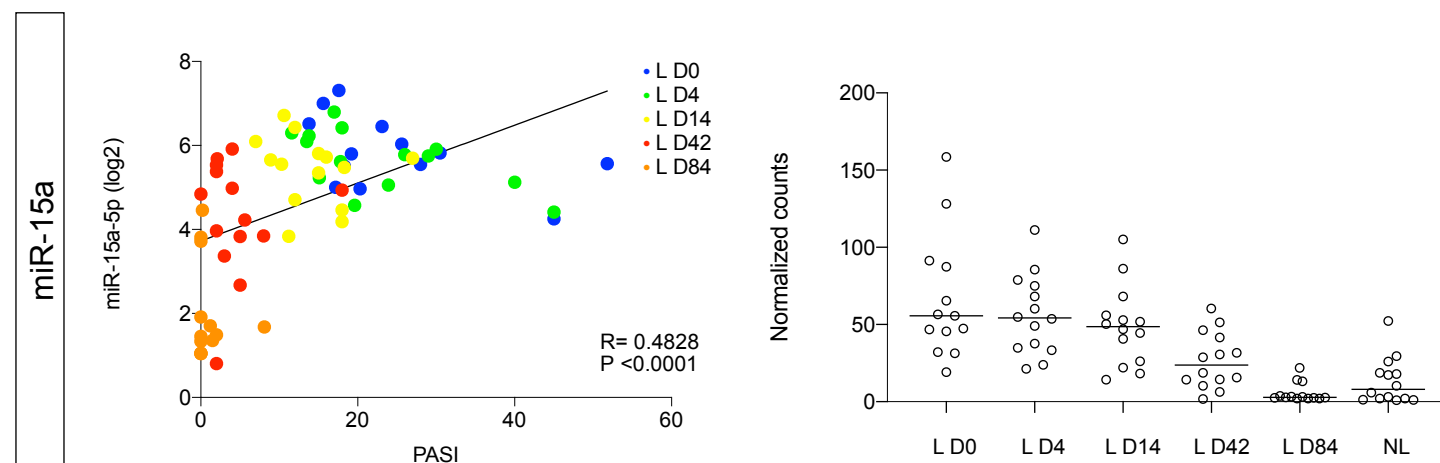

Figure S4

Supplement: S4 Fig — (A+B; left) CircTULP4 (A), miR-223-3p (B), and miR-15a-5p (C) log2-transformed expression values plotted against PASI during 84 days of secukinumab treatment. Simple linear regression was used for correlation between log2-transformed normalized counts and PASI. (A+B; right) CircTULP4 (A), miR-223-3p (B), and miR-15a-5p (C) expression in lesional skin during secukinumab treatment and non-lesional skin. Depicted are normalized counts and median expression; n(NL, L D4, L D14, and L D43) = 14, n(L D0) = 13, n(circRNA-L D84) = 12, and n(miRNA-L D84) = 13. (PDF) [file pone.0275219.s004.pdf]
