# Supplementary material for: Global circRNA expression changes predate clinical and histological improvements of psoriasis patients upon secukinumab treatment
Source: PLoS One. 2022 Sep 29;17(9):e0275219. doi: 10.1371/journal.pone.0275219 (PMC9522259; doi:10.1371/journal.pone.0275219)
Supplement: Attachment — Submitted filename: PONE-D-22-15625_review.pdf.docx [file pone.0275219.s013.docx]

The original research work of Kristensen’s Laboratory “Global circRNA expression changes predate clinical and histological improvements of psoriasis patients upon secukinumab treatment” has shown the involvement of non-coding RNA and, in particular circular RNA, in psoriasis pathogenesis and a possibility to normalize the level of a set of circRNA in lesional skin by secukinumab, the first targeted anti-IL-17A drag for psoriasis treatment. These priority findings open opportunities for the use of circRNAs as predictive markers for treatment efficacy and as prognostic markers for disease progression. Moreover, it reveals novel approaches for the treatment of psoriasis directed to normalizing of ncRNA imbalances. Design of the study is well done and the methods used are perfectly corresponded to the aims. All the protocols for experimental procedures are provided in details. Modern statistical analysis has been applied for the data processing. The results have been described and discussed properly and well presented in figures and tables. Some data of the study, especially concerning micro RNA dynamics during psoriasis progression, are in accordance with the previously published one, and the whole concept of the study is in agreement with current knowledge of circular RNAs. Thus, this work may be recommended for acceptance for publication in PLOS One journal as the original research article. However, some minor revision of the text is required:

1. Authors should check whether all the abbreviations are included in the text. As an example, there is no abbreviation for peripheral blood mononuclear cells – PBMC.
2. There is correct description of the histologic slide preparing, however there is a mistake in the Figure 2 legend (hematoxylin-eosin staining is pointed out).
3. There is a typing error on the page 7, line 1: it should be “All **p**-values” instead of “All **P**-values”.
4. The authors may discuss correlation of their data with the results of Bertelsen et al., 2020 concerning visible Ki67 staining up to 14 day of the treatment with secukinumab, but disappearance of Ki67 staining since 42 day of secukinumab treatment (ref. 13).
5. Pairing of the groups provided in the figures should be done more carefully as it should correspond to the logic of the study, for example in the figure 1 the columns NL and L D84 should change places. I would recommend to check all the figures relatively to this point.
